# Estrous cycle dependent expression of oxycodone conditioned reward in rats

**DOI:** 10.1038/s41598-023-40971-3

**Published:** 2023-08-25

**Authors:** Jessica A. Babb, Nicholas J. Constantino, Gary B. Kaplan, Elena H. Chartoff

**Affiliations:** 1 Research and Mental Health Services, VA Boston Healthcare System, Boston, Massachusetts USA; 2 Department of Psychiatry, Harvard Medical School, Boston, Massachusetts USA; 3 Division of Basic Neuroscience, McLean Hospital, Belmont, Massachusetts USA; 4 Department of Psychiatry, Boston University School of Medicine, Boston, Massachusetts USA

**Keywords:** Reward, Motivation, Addiction

## Abstract

Oxycodone is one of the most widely prescribed and misused opioid painkillers in the United States. Evidence suggests that biological sex and hormonal status can impact drug reward in humans and rodents, but the extent to which these factors can influence the rewarding effects of oxycodone is unclear. The purpose of this study was to utilize place conditioning to determine the effects of sex and female hormonal status on the expression of oxycodone conditioned reward in rats. Gonadally intact adult Sprague-Dawley male and female rats were used to test: (1) whether both sexes express conditioned reward to oxycodone at similar doses, (2) the impact of conditioning session length on oxycodone conditioned reward expression in both sexes, and (3) the influence of female estrous cycle stage on oxycodone conditioned reward expression. Both sexes expressed conditioned reward at the same doses of oxycodone. Increasing the length of conditioning sessions did not reveal an effect of sex and resulted in lower magnitude conditioned reward expression. Importantly however, female stage of estrous cycle significantly influenced oxycodone conditioned reward expression. These results suggest that female hormonal status can impact the rewarding effects of opioids and thus have important implications for prescription opioid treatment practices.

## Introduction

Although efforts have been made to curb over-prescribing of opioid analgesics, the opioid epidemic remains an ongoing public health crisis in the United States. The non-medical misuse of prescription opioids has been decreasing in recent years, but a concurrent increase in the use of heroin has been observed^1^. Within these trends, misuse of prescription opioids has been decreasing less rapidly, and heroin use increasing more rapidly, in women compared to men^2^. As a result, women are now just as likely as men to use heroin and to misuse prescription opioids^3–6^. Women tend to have more severe and more chronic pain than men^7^ which could explain their greater misuse of prescription opioids, but women are also more likely to use prescription opioids to cope with stress, anxiety, and depression^8, 9^. Psychiatric disorder diagnoses are strongly associated with overdose deaths from prescription drugs^10^, and women with opioid dependence are more likely to have mood and anxiety disorders than men^11^. More research is needed to better understand the behavioral and neurobiological consequences of opioid use in females that could impact the development and progression of, as well as the recovery from, opioid use disorder^12, 13^.

Clinical research studies suggest that men and women experience different trajectories of substance use disorders. For example, although in general more men than women use drugs and alcohol, women tend to progress from initial drug use to dependence and substance use treatment faster than men for a wide variety of substances including opioids^14–16^. Women also tend to experience more adverse outcomes with drug use than men, including more severe withdrawal, greater cravings, higher propensity for relapse, and poorer overall psychological and physical health^17–19^. It is still unclear, however, to what degree these observed differences are biologically based as opposed to being driven by cultural factors; for instance, the impact of biological sex on drug and alcohol use can vary across factors such as age.

Results from preclinical rodent studies have mirrored what is observed in humans for some substances, but not others. In early rodent studies that primarily used psychostimulants, females tend to acquire self-administration faster, escalate their drug intake more rapidly, and show greater preference for drug-paired contexts in place conditioning paradigms compared to males^15^. However, observed sex differences in drug seeking for other substances, such as opioids, are less consistent and can vary across important but often overlooked experimental factors such as strain, drug dose, and time of day^15^. For example, female Wistar rats showed higher morphine-induced conditioned place preference (CPP) than male Wistar rats at doses less than 10 mg/kg^20^, but two other studies found no effect of sex on morphine CPP in Sprague-Dawley rats at doses less than 10 mg/kg, although females showed higher morphine CPP than males at doses of 10 mg/kg or higher^21, 22^. Rodent studies evaluating sex differences in CPP for the prescription opioid oxycodone have been similarly inconsistent, with reports of greater oxycodone CPP in females^23^, and other reports of no significant effect of sex^24, 25^. Several methodological factors, such as dose and conditioning session duration could mediate the impact of sex on the expression of CPP. The experiments in the current study investigate the impact of such factors on the expression of oxycodone reward in adult male and female Sprague-Dawley rats using place conditioning^26^.

A critical factor that broadly affects drug reward is female hormonal status^16^. For example, in humans, menstrual cycle phase can impact the subjective effects of drugs^27, 28^ as well as withdrawal severity, drug craving and relapse vulnerability among women with substance use disorders^15, 29–31^. In female rodents, estrous cycle phase has been shown to affect drug-motivated behaviors across a wide range of substances, including opioids^15, 32^. For example, heroin self-administration is influenced by the estrous cycle^33, 34^. Therefore, in this study we also investigated the influence of estrous cycle phase on oxycodone conditioned reward. To our knowledge, there are no published studies to date that have investigated the impact of estrous cycle or specific hormones on oxycodone-motivated behaviors using place conditioning.

## Results

### Effect of conditioning session length on oxycodone place conditioning

**Figure 1:**
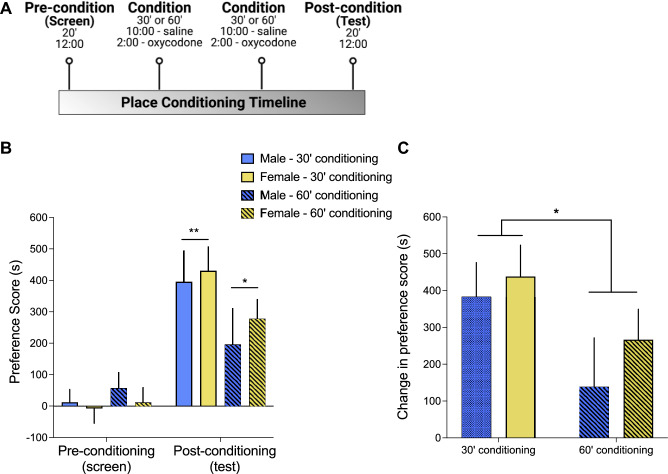
Effect of conditioning session length on oxycodone conditioned place preferences in male and female rats. (**A**) Experimental design schematic depicting timing of testing over 4 consecutive days. Rats were conditioned with oxycodone (3.0 mg/kg, s.c.) for either 30 min (solid bars in B/C) or 60 min (hatched bars in B/C) over 2 days and place preferences were tested for 20 min on the days preceding (screen) and following (test) the two conditioning days. Data (means ± SEM) are expressed as (**B**) preference scores (calculated as the amount of time spent on the oxycodone-paired side minus the time spent in vehicle-paired side during pre- and post-conditioning test sessions), and (**C**) the change in preference score (post-conditioning minus pre-conditioning score). *significant difference, p < 0.05; **p < 0.01. n = 8 rats/group.

Using a 4-day place conditioning paradigm (Fig. 1A), we first compared oxycodone place conditioning in adult male and female rats after two lengths of conditioning sessions (30 vs. 60 min in duration). These two durations of conditioning were chosen based on the known acute transient sedative effects of oxycodone^35^, which we have observed to last approximately 30 min in rats (unpublished observations). Comparing pre- and post-conditioning preference scores (calculated as the time spent in the oxycodone-paired context minus the time spent in the saline-paired context) using repeated measures ANOVA revealed that oxycodone induced robust place preferences, as indicated by significantly greater preference scores in the post-conditioning test compared to pre-conditioning screen (significant main effect of conditioning; F_1,28_ = 37.3, p < 0.001; Fig. 1B). Oxycodone CPP was not affected by sex (no main effect of sex or interaction between sex and time or conditioning length) but was dependent on length of conditioning session (significant interaction between time and conditioning duration; F_1,28_ = 4.3, p = 0.047; Figure 1B). Post-hoc paired samples t-tests comparing pre- and post-conditioning preference scores for each conditioning session length revealed significant oxycodone CPP after both 30-min (t_15_ = 6.71, p < 0.001) and 60-min (t_15_ = 2.62, p = 0.02) conditioning sessions (Fig. 1B). However, conditioning sessions that were 30 min in duration resulted in higher CPP compared to 60-min sessions. Specifically, the change in preference score from the pre-conditioning screen to the post-conditioning test was significantly higher after 30 min conditioning sessions than after 60 min conditioning sessions (F_1,28_ = 4.3, p = 0.047; Fig. 1C). Regardless of conditioning session length, there was no significant impact of sex on oxycodone conditioned reward (no main effect of sex or significant sex interaction, p’s > 0.05).

### Oxycodone place conditioning dose-response curve

**Figure 2:**
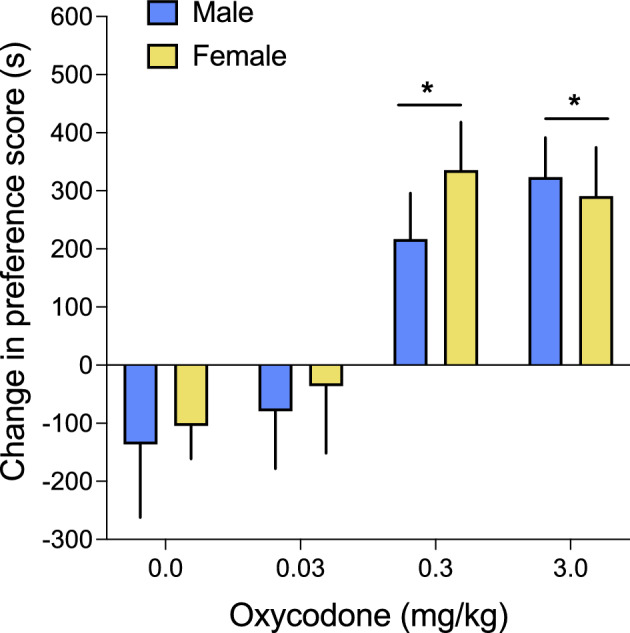
Oxycodone dose-dependently induces conditioned place preference in adult male and female rats. Rats were conditioned with 0.0 (n = 4/sex), 0.03 (n = 7–8/sex), 0.3 (n = 15–16/sex), or 3.0 (n = 16/sex) mg/kg oxycodone in 30-min sessions on two consecutive days (see Fig 1A). Oxycodone (0.3 and 3.0 mg/kg) induced significant conditioned place preference in the test session (*significantly higher than vehicle-treated rats (saline; 0.0 mg/kg), p < 0.05, 2-way ANOVA). Data are expressed as the change in preference score (means ± SEM).

Figure 2 displays the dose-response function for oxycodone place conditioning at increasing doses (0–3 mg/kg, s.c.) in male and female rats. A two-way ANOVA revealed a significant effect of dose on the change in preference score (from pre-conditioning screen to post-conditioning test; calculated as time spent on the oxycodone-paired side minus time spent on vehicle-paired side; F_3,78_ = 8.59; p < 0.001; Fig. 2). Post-hoc testing revealed significant conditioned place preference at the two highest doses (0.3 and 3.0 mg/kg oxycodone) compared to rats that received vehicle (0.0 mg/kg; saline) or 0.03 mg/kg oxycodone (p’s < 0.05). The change in preference score was similar in rats that received 0.3 or 3.0 mg/kg oxycodone, and rats that received the lowest dose of oxycodone (0.03 mg/kg) did not differ significantly from rats that received saline vehicle injections (p’s > 0.05). The dose-response function did not differ between male and female rats, as evidenced by the lack of main effect of sex or significant interaction between sex and oxycodone dose on the change in preference score (p’s > 0.05).

### Impact of sex and estrous cycle stage on oxycodone conditioned reward

**Figure 3:**
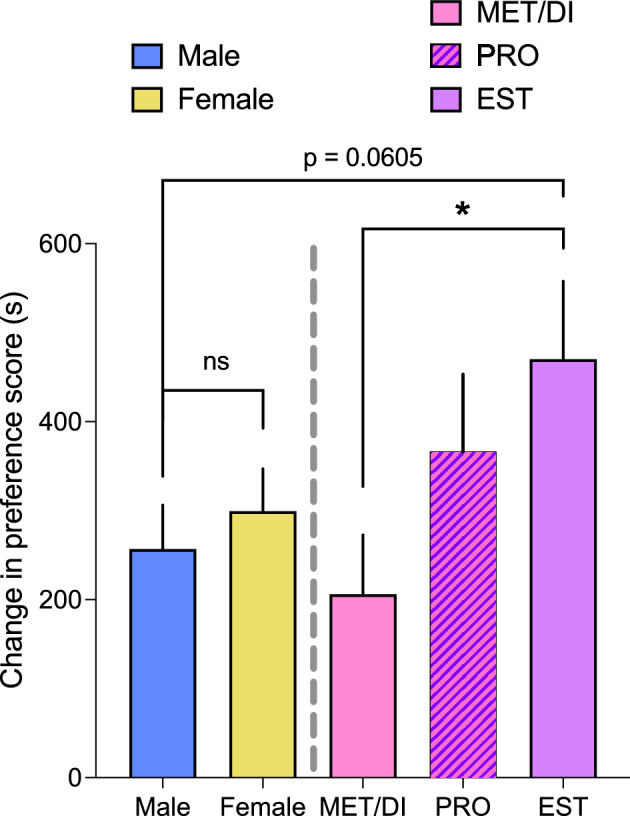
Oxycodone induces greater conditioned place preference in female rats during estrus. Oxycodone (3.0 mg/kg) induced similar significant place preferences in adult male and female rats when estrous cycle stage is not factored; n = 38 rats/sex, left panel. When estrous cycle on the post-conditioning test day could be accurately determined (35 out of 38 females), oxycodone place conditioning was significantly higher when rats were in estrus (EST; n = 9) compared to rats in either metestrus or diestrus (MET/DI; n = 22) or rats in proestrus (PRO; n = 4). Data (group means + SEM) are expressed as the change in preference score (post-conditioning minus pre-conditioning). *significant difference, p < 0.05. *ns* not significantly different.

To investigate the impact of estrous cycle phase on the expression of oxycodone conditioned place preference, a large cohort of male and female rats (a total of 38 rats of each sex) were place conditioned with parameters which produced the greatest preference for the oxycodone-paired side in the first two experiments (30 min conditioning sessions with 3 mg/kg oxycodone). A repeated measures ANOVA revealed that place conditioning with 3 mg/kg oxycodone induced a robust preference for the oxycodone-paired context in both sexes (significantly greater time spent on oxycodone-paired side during post-conditioning test compared to pre-conditioning test; F_1,74_ = 64.99, p < 0.001). No effect of sex was observed on the change in preference scores from pre-conditioning screen to post-conditioning test (no main effect of sex or interaction between sex and conditioning on preference scores, p’s > 0.05; Fig. 3, left panel).

We then analyzed the estrous cycle of each female rat to determine each rat’s stage on the post-conditioning test day. Three out of thirty-eight females were not cycling normally and were eliminated from further analyses. Of the remaining 35 females, 12 females were in metestrus (MET), 10 were in diestrus (DI), 4 were in proestrus (PRO), and 9 were in estrus (EST) on the post-conditioning test day. The change in preference score was statistically similar between females in metestrus or diestrus (MET females: mean of 179, SEM 95; DI females: mean of 239, SEM 98). The hormone profile of females in metestrus and diestrus are very similar and these stages are sometimes referred to as Diestrus I and Diestrus II. Therefore, data from these rats were combined to a group of 22 females that were in either metestrus or diestrus (MET/DI) on the test day. Females that were in EST had a significantly greater change in preference score than MET/DI females (t_29_ = 2.2, p = 0.03; Fig. 3, right panel). The change in preference scores in the 4 proestrus females did not differ significantly from either MET/DI females (t_24_ = 0.98, p> 0.05) or from females in estrus (t_11_ = 0.71, p > 0.05). Although females in estrus had a larger change in preference score than males, this difference did not reach statistical significance (t_45_ = 1.93, p = 0.06; Fig. 3).

**Figure 4:**
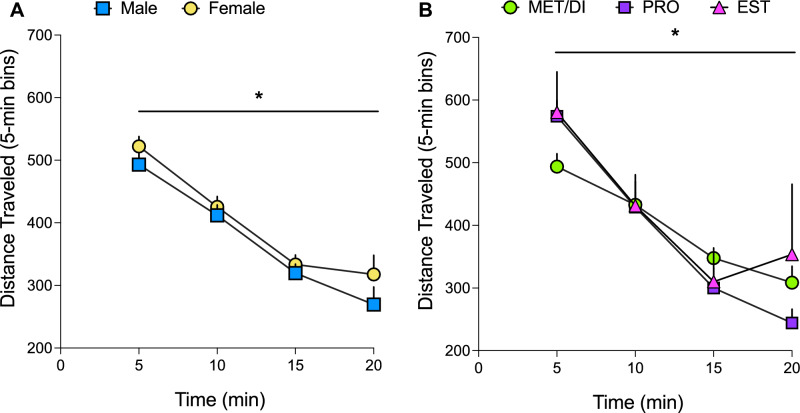
Locomotor activity during the post-conditioning test session did not vary by sex or by estrous cycle stage in female rats. Locomotion (group means + SEM) during the 20-min post-conditioning test session is displayed as activity in 5-min bins in (**A**) males and females when estrous cycle is not factored (n = 38/sex) and (**B**) female rats in EST (n = 9), PRO (n = 4), and MET/DI (n = 22) on the test day. *significant main effect of time, p < 0.05.

Over the course of the 20-min post-conditioning test, the amount of time spent in the oxycodone-paired context gradually increased while time spent in the vehicle-paired context gradually decreased, but the pattern of these changes did not vary by estrous cycle stage (p’s > 0.05; data not shown). Total locomotor activity during the 20-min post-conditioning test did not differ significantly between males and females, or between MET/DI, PRO, or EST groups (p’s > 0.05). Dividing the 20-min post-conditioning test into 5-min bins revealed that locomotor activity significantly decreased over the course of the 20-min post-conditioning test for males and females overall (F_1.87, 138.6_ = 63.23, p < 0.001; Fig. 4A) and for MET/DI, PRO, and EST females (F_1.8, 57.7_ = 24.97, p < 0.001; Fig. 4B), but importantly this decrease in activity was similar across sexes and estrous cycle stages (no main effect or interaction of sex/estrous cycle, p’s > 0.05; Fig. 4).

## Discussion

Evidence suggests that the adverse consequences of opioid misuse are greater in women than in men^14–18^. Despite this clinical evidence, research using rodent models to study the effects of opioid have largely omitted female subjects, and even fewer have systematically evaluated the impact of fluctuating hormones^12^. In this study we investigated the effects of biological sex and female estrous cycle phase on the rewarding effects of the prescription opioid oxycodone using a 4-day place conditioning paradigm in adult male and female rats. We found that when estrous cycle stage is not considered, male and female rats do not differ in the expression of oxycodone reward using a place conditioning paradigm in response to varying conditioning session durations (30 vs 60 min) and across a range of doses (0.03, 0.3, and 3 mg/kg). However, we found that estrus females had significantly higher oxycodone CPP than metestrus and diestrus females, indicating that the estrous cycle influences the expression of oxycodone reward in female rats. These results suggest that the rewarding effects of oxycodone in females depend on fluctuating gonadal hormone concentrations. Importantly, in the absence of factoring in female estrous cycle stage, oxycodone CPP is similar between male and female rats, which engenders incomplete and inaccurate conclusions that have ramifications for the treatment of opioid use disorder in women.

In this study oxycodone induced a significant CPP for the oxycodone-paired compartment in both males and females at doses of 0.3 mg/kg and 3.0 mg/kg oxycodone, but not at a dose of 0.03 mg/kg (Fig. 1). These results are mainly consistent with previous reports. For example, CPP was previously observed in adult male rats following oxycodone conditioning at 1 and 3 mg/kg doses, but not at a dose of 0.3 mg/kg^36^. Likewise, Collins and colleagues^24^ observed oxycodone CPP in both male and female C57BL/6J mice at doses of 1, 3, and 10 mg/kg but not at a dose of 0.3 mg/kg. One reason for the discrepant findings at the 0.3 mg/kg dose could be differences in the route of oxycodone administration (i.p. in the aforementioned studies vs. s.c in the current study). Consistent with this idea, Liu and colleagues^37^ observed significant CPP in Wistar rats (sex unknown) after conditioning with oxycodone injected s.c. at doses of 0.32, 0.625, 1.25, 2.5, and 5.0 mg/kg (but no CPP was observed at a dose of 0.16 mg/kg oxycodone). CPP has also been observed previously after conditioning with s.c. injections of 3 mg/kg oxycodone in female Sprague-Dawley rats^38^. Similar to the observations of Rutten and colleagues^36^ though, we did not observe dose dependency between doses that induced CPP (i.e., the magnitude of CPP did not differ between the 0.3 mg/kg and 3.0 mg/kg doses of oxycodone).

A meta-analysis of rodent studies of opioid and stimulant CPP determined that there is likely an inverted U-shaped relationship between duration of conditioning sessions and CPP effect sizes^39^. However, the conditioning session length that produces peak CPP can vary depending on the opioid studied, which may reflect the specific pharmacokinetics and/or pharmacodynamics of that opioid. For example, larger effect sizes of heroin CPP were observed when conditioning trials were 25–30 min in duration compared to 20 min or less, but for morphine CPP, greater effect sizes were observed when conditioning trials were 45 min or more, compared to conditioning trials that were 25–30 min in duration^39^. In the current study, both durations of place conditioning sessions (30 vs. 60 min) significantly induced CPP in both sexes. Contrary to our expectation, the magnitude of CPP was significantly higher in the shorter duration (30 min) conditioning group (Fig. 2). It can be challenging to directly compare opioid pharmacology across studies of different opioids, as variables such as route of drug administration can impact opioid pharmacokinetics. Nevertheless, intravenous injection of oxycodone is known to lead to a rapid increase in plasma concentrations (peak at 5 min in both sexes), and both plasma and brain concentrations of oxycodone are similar after i.v. injection in male and female rats^40^, suggesting that oxycodone pharmacokinetics do not differ by sex. Our results show that at least for oxycodone injected s.c., conditioning sessions of 30 min lead to more effective conditioning than sessions of 60 min in duration, and this was true for both sexes. It is therefore likely that a conditioning session duration of 60 min is beyond the peak of the inverted U-shaped curve for oxycodone CPP effect size. It is possible that higher oxycodone CPP effect sizes could be observed with conditioning session durations between 30 and 60 min, since the pharmacological effects of oxycodone are similar to those of morphine^35^, which produces maximal CPP after 45-min conditioning sessions^39^.

Previous studies conflict regarding whether sex differences in oxycodone conditioned reward exist^23–25^. Here, we tested an important factor that could impact whether (or not) sex differences in oxycodone CPP are observed: the hormonal state of female subjects. In humans, the menstrual cycle has been demonstrated to affect drug use in women^15, 29–31^. Preclinical studies have provided evidence that the rodent estrous cycle can also impact drug-seeking^15, 32^, as well as voluntary intake of opioids^33, 34^. When we tested oxycodone CPP under maximal parameters (30 min conditioning sessions with 3 mg/kg oxycodone) in a large cohort of male and female rats to reduce behavioral response variability and increase statistical power, oxycodone robustly induced CPP in both sexes (Fig. 3). When we then split female rats based on the estrous cycle stage on the test day, we observed significantly greater oxycodone CPP expressed in females during estrus compared to metestrus and diestrus phases of the estrous cycle (Fig. 3). Female rats in proestrus on the test day expressed a similar magnitude of oxycodone CPP as female rats in estrus but did not express significantly higher CPP than female rats in metestrus or diestrus. The lack of statistical power due to the small sample size of females in proestrus on the test day (n = 4) makes it hard to draw any strong conclusions about the expression of conditioned reward in this group. Females in estrus on the test day had higher oxycodone conditioned reward than males, although this difference did not reach statistical significance. Nonetheless, these data suggest that conflicting effects of sex observed in prior studies of opioid conditioned reward could be due to the influence of estrous cycle on CPP expression.

Collectively, our results suggest that female rat hormonal state (i.e. fluctuating steroid hormones) can influence the expression of oxycodone conditioned reward. In human studies, drugs generally appear to be more appetitive in the follicular and ovulatory phases (when estrogens are relatively high) compared to the midluteal phase of the menstrual cycle (when progesterone is relatively high^29, 31^). Higher drug craving and shorter relapse latency are also associated with the late follicular/ovulatory phases of the menstrual cycle and conversely higher withdrawal severity and decreased subjective reward are associated with the midluteal phase^30^. Rodent studies (mainly using cocaine) have also supported the idea that estrogens enhance drug-motivated behaviors, whereas progesterone dampens drug responses. However, studies that have evaluated the effect of specific hormones on opioid-motivated behaviors in ovariectomized rodents are inconclusive. For example, Stewart and colleagues^41^ found no effect of estradiol benzoate on heroin self-administration in ovariectomized rats, but Roth and colleagues^42^ found that ovariectomized rats treated with estradiol benzoate acquired heroin self-administration significantly faster than vehicle-treated controls. And very recently, Smith and colleagues^34^ found that ovariectomized female rats treated with estradiol self-administered significantly less heroin than those treated with progesterone, which may indicate heightened sensitivity to heroin after estradiol treatment. To our knowledge, no studies to date have investigated the impact of specific hormones on oxycodone-motivated behaviors (i.e. using place conditioning or self-administration models).

Ovulation occurs in the dark phase of the light cycle between the proestrus and estrus stages of the estrous cycle^43, 44^. Proestrus and estrus have markedly different profiles of estradiol, which rise across the light phase of proestrus to peak at ovulation during the dark phase, and then drop to very low levels by the beginning of the estrus light phase^44, 45^. In contrast, progesterone levels are very low on both the mornings of proestrus and estrus, spike rapidly in the dark phase between these two stages and are moderately higher and relatively stable during metestrus and diestrus^45^. Therefore, it is likely that estradiol levels differed substantially during the light phases of proestrus and estrus (when we conducted our testing), but progesterone levels may have been similarly low in these two phases compared to metestrus and diestrus. It is thus possible that progesterone has an inhibitory effect on the expression of oxycodone conditioned reward, which would fit with both preclinical and clinical findings of the role of hormones on drug-motivated behavior^31, 32^. Another possibility is that the rapid fluctuation of hormones from proestrus to estrus leads to increased oxycodone conditioned reward compared to MET/DI, when hormones are relatively stable. More research is needed to test the temporal effects of specific hormones on oxycodone behaviors.

One caveat of these interpretations, however, is that the phase of estrous cycle on the post-conditioning test day is not the causal factor in the CPP differences we observed since the administration of the drug occurred on the two prior consecutive days to the post-conditioning test day. Other types of motivated behavior (i.e., voluntary wheel running) have been observed as significantly higher in females during proestrus and estrus compared to metestrus and diestrus^46^, which is in line with the estrous cycle effect we observed here. Higher drug-seeking behavior specifically during the estrus stage has been observed after cocaine, nicotine, or fentanyl self-administration^47–49^. These studies suggest that drug craving is impacted by hormonal state and is highest during estrus. However, Calipari and colleagues^50, 51^ have demonstrated that increased motivation is observed when drug-associated cues are paired with drug experience during estrus, suggesting that later motivation is driven primarily by cue-associations developed under the influence of drug itself. Another interpretation of our findings is that estrous cycle/hormonal state influences the recall of conditioned context. Evidence that supports this idea is that contextual cued recall of prior conditioned fear has been shown to be impacted by stage of estrous cycle^52^. One way to test between these interpretations on the effects observed here would be to conduct conditioning sessions and preference tests during the same stage of the estrous cycle, or to test the expression of conditioned reward after varying lengths of time after conditioning sessions (i.e., 48 h after the last session vs. 24  h after) to see if a similar effect of estrous cycle is observed.

In summary, these experiments investigated potential effects of sex and estrous cycle on the rewarding effects of the prescription opioid oxycodone using a place conditioning paradigm. Our results demonstrate that oxycodone is robustly rewarding to both sexes. Importantly, we also demonstrate that female estrous cycle stage significantly influences the expression of oxycodone conditioned reward. These results suggest that female hormones can modify the expression of oxycodone reward and could contribute to conflicting observations of sex differences in opioid-seeking studies. These experiments therefore highlight the importance of considering the influence of female hormonal state—a translationally relevant and yet often overlooked factor—on opioid-motivated behaviors. Finally, these findings could have important implications for abuse liability for women who are prescribed opioids such as oxycodone for analgesia.

## Methods

### Subjects

A total of 151 adult Sprague-Dawley rats (CD strain; Charles River Laboratories, Wilmington, MA) were utilized in these experiments (N = 76 male; 275-300 g and N = 75 female; 200–225 g). Upon arrival, rats were housed in same-sex groups in standard rectangular cages on a 12 h light/dark cycle (lights on at 7 am) with food and water available *ad libitum*. Rats were acclimated to the colony room for at least 1 week prior to experimental manipulation. All experimentation was conducted during the light phase, in accordance with the guidelines of the National Institutes of Health “Guide for the Care and Use of Laboratory Animals” and the American Veterinary Medical Association’s 2020 “Guidelines for the euthanasia of animals”. All procedures were reviewed and approved by the Institutional Animal Care and Use Committee at McLean Hospital and are reported in compliance with the ARRIVE guidelines.

### Handling and estrous cycle monitoring

Female estrous cyclicity was monitored by collecting daily vaginal lavage samples before 10 am in a procedure room separate from the housing colony beginning the week prior to behavioral testing. Males were handled daily in a similar manner to control for this procedure (handled by base of tail for a similar amount of time in the same separate procedure room concurrent with lavage collection). Lavage samples were collected with glass eye droppers containing 0.9% saline and dried on Superfrost Plus slides (Fisher Scientific) overnight, after which slides were stored until being stained with Harris hematoxylin and Eosin Y, as described previously^45^. Stage of estrous cycle was determined by light microscopic assessment of epithelial cytology in lavage samples. For the estrous cycle experiment, only female rats that displayed normal 4–5 day estrous cycles and clear cycle stage on the test day were included in analysis.

### Apparatus

Place conditioning was conducted in commercially available chambers with 3 compartments (Med Associates, St. Albans, VT). The two larger equal sized compartments were separated by a smaller middle compartment by manual doors and were distinguishable by both visual and tactile cues. One large compartment consisted of white walls and a wire mesh grid floor, whereas the other large compartment had black walls and a floor with wider parallel bars. The middle compartment consisted of a solid smooth floor with all gray surfaces (walls and floor). All 3 compartments were equipped with overhead lights and infrared photobeam detectors located just above the floors. The amount of time spent and locomotor activity within each compartment were tracked by beam breaks that were automatically recorded and sent to a computer located in an adjacent room. The intensity of the overhead lights in each of the 3 compartments were adjusted until there was no statistically significant difference in the amount of time spent in each larger chamber (i.e. no baseline bias in compartment preference, tested using separate groups of rats prior to any of the currently described experiments, data not shown).

### Oxycodone place conditioning

Experiments were conducted over 4 consecutive days in 3 phases (Screening, Conditioning, and Testing; Fig. 1A). On all experimental days, rats were transported within their home cages to a room adjacent to the room housing the conditioning chambers, where lavage samples were taken and body weights were recorded, after which rats were left undisturbed in their home cages for at least 20 min prior to manipulation. In the screening phase, each rat was placed in the middle compartment with doors open to allow free access to the entire apparatus, and locomotor activity was monitored for 20 min. The amount of time spent in each of the three compartments was measured. Any rat that exhibited a bias for one compartment over another (defined as more than twice the total amount of time spent in one of the large compartments) was excluded from further behavioral testing (2 out of the total 151 rats tested were excluded, both were female). During the conditioning phase (2 days total) rats were injected with vehicle (0.9% saline; s.c.; injection between 10 and 11 am) and were then immediately confined to one of the 2 large compartments of the apparatus (with doors closed to prevent access to middle compartment). In the afternoon, rats were injected with oxycodone hydrochloride (dissolved in 0.9% saline; s.c; injection between 2 and 3 pm) and were confined to the opposite side of the apparatus. The assignment of the oxycodone-paired compartment was unbiased and counter-balanced such that half of each sex were assigned to oxycodone conditioning in each of the two larger compartments. For the conditioning length experiment, sessions were either 30 or 60 min in duration (n = 8/sex/conditioning duration). For all other experiments, conditioning sessions were 30 min in duration. For the dose-response experiment, rats received one of the following doses of oxycodone in the afternoon conditioning session: 0.0 (0.9% saline; n = 4/sex), 0.03 (n = 8/sex), 0.3 (n = 15–16/sex), or 3.0 mg/kg (n = 16/sex). For the conditioning length and estrous cycle experiments, all rats received the highest dose of oxycodone (3 mg/kg). During the testing phase, rats were again placed in the middle compartment with doors open to allow free access to the entire chamber for 20 min, and locomotor activity and time spent in each compartment were recorded. At the end of each day, rats were returned to their home cages and then transported back to the colony room.

### Data calculations and statistical analyses

Statistical analyses were performed, and graphics created, using GraphPad Prism (Version 9). Time spent in the oxycodone-paired compartment during both the screening and testing phases (Fig. 1A) were calculated for each rat by subtracting the total amount of time spent in the vehicle-paired compartment from the total amount of time spent in the oxycodone-paired compartment. The change in time spent on the oxycodone-paired side was further calculated by subtracting the pre-conditioning (screening) from the post-conditioning (test). For the dose response experiment, the change in time spent in the oxycodone-paired compartment at each dose were analyzed using analysis of variance (ANOVA) with sex and dose as between-subjects factors, with Tukey’s honestly significant difference (HSD) tests used for post-hoc analyses. For all other experiments, pre- and post-conditioning scores were first analyzed using a repeated measures ANOVA with time as a within-subjects factor and either conditioning session length, sex, group, or estrous cycle as between-subjects factors. Then the change in time spent in the oxycodone-paired compartment was further analyzed using a univariate ANOVA. Total locomotor activity was analyzed with univariate ANOVAs and locomotor activity with test sessions was analyzed with a repeated measures ANOVA.

## Data Availability

The datasets generated during the current study are available from the corresponding author on reasonable request.
